# The validity and reliability of a hydraulic resistance device for assessing resisted sprint time

**DOI:** 10.3389/fspor.2024.1386882

**Published:** 2024-07-25

**Authors:** Matic Sašek, Oskar Cvjetičanin, Nejc Šarabon

**Affiliations:** ^1^ Faculty of Health Sciences, University of Primorska, Izola, Slovenia; ^2^ Human Health, InnoRenew CoE, Izola, Slovenia; ^3^ Andrej Marušič Institute, University of Primorska, Koper, Slovenia; ^4^ Ludwig Boltzmann Institute for Rehabilitation Research, Vienna, Austria

**Keywords:** resisted sprints, sprint performance, hydraulic resistance, measurement device, validity, reliability

## Abstract

**Introduction:**

The aim of this study was to assess the validity and reliability of a hydraulic resistance device (HRD) for monitoring sprint split times under different loads within and between sessions.

**Methods:**

Three 20-m sprints with low (15 N), medium-low (40 N), medium-high (50 N), and high (130 N) HRD resistance levels (loads) were performed on two separate occasions 14 days apart. Twenty-four student athletes (24.8 ± 3.8 years) participated in the first session and 13 (24.1 ± 3.2 years) of them in the second session. Resisted sprints split times over a distance of 0–20 m (t_0–5_, t_0–10_, t_0–20_, t_5–10_, t_10–15_, t_15–20_) were measured simultaneously with magnetic incremental encoder embedded in the HRD and a system of single-beam timing gates.

**Results:**

The results showed acceptable to high within session (ICC_3,1_ = 0.91–0.99; CV = 0.92%–3.38%) and between session (ICC_3,1_ = 0.82–0.99; CV = 1.62%–4.84%) reliability of HRD for measuring all split times at all loads. The minimal detectable change between sessions ranged from 3.3% at high load to 9.9% at low load. The HRD systematically underestimated timing gates times at all loads (bias = 2.01–11.08%), yet good to excellent consistency was observed between the HRD and timing gates, specifically for t_0–10_ and t_0–20_ (ICC_3,k_ lower 95% CI = 0.84–0.99).

**Discussion:**

Due to high reliability and good validity in monitoring resisted sprint times, the HRD holds potential for practical and research applications.

## 1 Introduction

Sprint and acceleration holds a pivotal role in determining success in a range of sports disciplines (1, 2). In addition to short-distance sprints (3), resisted sprints have garnered substantial attention for its ability to enhance acceleration speed acutely (4), as well as in the long-term (5–9). In the context of resisted sprinting, a force acts in the opposing horizontal direction to the movement of the sprinter's center of mass, commonly referred to as the resistance force. This necessitates athletes to exert substantial horizontal propulsive forces against the ground (10). Resisted sprint training improves horizontal force production capacity, which is one of the main kinetic indicators of sprint performance (11). Purposely, various devices are employed in practical settings to perform resisted sprints, including aerodynamic (e.g., running parachutes), motorized, pulley, or friction resistance (e.g., sprint sleds) (12, 13).

Non-motorized devices and assemblies designed for resistance sprinting commonly fall short in measuring sprint speed and the associated resistance force, critical components for tailoring training loads individually (12). Despite some successful attempts to estimate resistance force through the friction of sleds in prior studies (14, 15), these calculations are susceptible to a multitude of uncontrollable variables, resulting in variability in recorded resistance force. Furthermore, supplementary equipment, such as timing gates, lasers or radars, must be applied to measure sprint performance accurately in many cases when using resisted sprints assemblies. The variability in resistance force production and challenges in resisted sprint performance measurement on the field could limit the ability to individualize training loads when employing pulley, aerodynamic or friction resistance devices. Because the individualization of resistance has been found to be an important factor for efficient resisted sprint training (16, 17), motorized devices have been recently used, offering the dual benefits of generating and monitoring the resistance force while simultaneously measuring sprint speed (18, 19). The combination of resistance force with spatiotemporal data of sprint forms the foundation for calculating the force-velocity profile (19, 20) and the load-velocity relationship (17, 21, 22). These two outcomes enable the prescription of specific and individualized training loads (23, 24).

Despite the prevalent use of resisted sprints in variety of cyclic movements such as running, skating (25), and swimming (26), the literature on the methodological aspects of non-motorized (i.e., mechanical or pulley) devices for resisted sprints is scarce. Moreover, the comprehensive understanding of resistance force and sprint performance when utilizing hydraulic resistance is lacking. In the present study, we introduce a novel solution, hydraulic-resistance based device (HRD), that produces isotonic resistance force while sprinting. Our unpublished investigations already confirmed consistency of resistance provided by a HRD, therefore the aim of this study was to additionally quantify its ability to assess sprint performance. Our objectives encompass the assessment of within and between session reliability and validity of HRD for measuring sprint split times. We hypothesized HRD would exhibit a high degree of validity and reliability in the measurement of sprint performance. Devices capable of real-time sprint time monitoring that provide objective resistance force can be used to individualize resisted sprint loads on the field, therefore the result of this study would provide valuable information for sports practitioners and athletes striving to enhance speed performance.

## 2 Materials and methods

### 2.1 Experimental approach to the problem

A cross-sectional study with two visits was conducted. During each visit, participants completed 20-m sprints with HRD load of 15 N (low), 40 N (medium-low), 50 N (medium-high), and 130 N (high) of resistance force. Sprint split times [at distances from 0 to 5 (t_0–5_), 0 to 10 (t_0–10_), 0 to 20 (t_0–20_), 5 to 10 (t_5–10_), 10 to 15 (t_10–15_), and 15 to 20 m (t_15–20_)] were simultaneously measured with the HRD and single-beam timing gates under all loads.

### 2.2 Subjects

A sample size power for the intraclass correlation coefficient (ICC) was *a priori* calculated. For minimum acceptable reliability of 0.5, expected reliability of 0.9 (18), a significance level of 0.05, a statistical power of 0.8, and 2 test-retest trials, a total of 11 participants was required (27). For that purpose, 24 student athletes participated at the first visit and 13 of them were recruited for the second visit. Descriptive statistics of subjects are reported in Table 1. All subjects were amateur athletes from different sport disciplines (i.e., martial arts, football, handball, basketball, track and field, and hockey), involved in training regime consisting of at least 6 sport-specific training sessions per week. Subjects did not report any chronic disease or a recent injury that could compromise the outcomes of the present study. Subjects were informed of the study procedures and signed the informed consent prior the study. The study was conducted in accordance with the Declaration of Helsinki, reviewed, and approved by Medical Ethics Committee under the grant 0120-690/2017/8.

**Table 1:** Descriptive statistics (mean ± SD) of subjects at the first and at the second session.

	First session			Second session		
	Male (*n* = 9)	Female (*n* = 15)	Total (*n* = 24)	Male (*n* = 4)	Female (*n* = 9)	Total (*n* = 13)
Age	26.8 ± 3.2	23.7 ± 3.6	24.8 ± 3.8	25.0 ± 3.2	23.9 ± 3.5	24.1 ± 3.2
BH (cm)	181.4 ± 5.1	166.5 ± 7.9	172.1 10.1	179.5 ± 3.1	170.2 ± 7.5	173.8 ± 7.9
BM (kg)	80.4 ± 6.4	63.8 ± 4.7	69.8 ± 10.0	76.8 ± 5.9	63.4 ± 5.1	68.4 ± 8.5

BH, body height, BM, body mass.

### 2.3 Procedures

**Figure 1:**
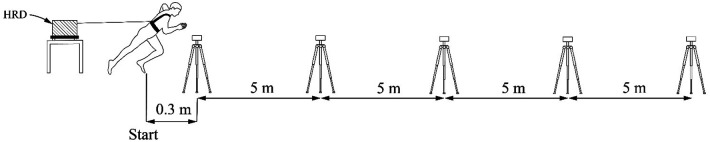
The setup for the resisted sprints. The timing gates were positioned on 20-m distance, 5 m apparat. The hydraulic resistance device (HRD) was positioned on rigid box, approximately 2 meters behind start line. Sprint start was performed with the front foot positioned 30 cm prior to first pair of timing gates. The HRD was attached to the subject via harness by using special designed belt.

Two identical testing procedures were repeated 14 days apart in the gym with a wooden sports floor. A standardized warm-up procedure included 4 min of 50-cm box stepping at a rate of 100 beats per minute, dynamic gymnastics exercises, lower-limb strength exercises (heel raises, hip raises, squats, crunches, and push-ups; 10 repetitions each), running drills (skipping, high knees, hopping, etc.), and three 20-m submaximal sprints. After the warm-up, three maximal sprints with four levels of resistance were performed in a balanced and randomized order. Resistance force during the sprints was provided by the HRD, positioned and firmly fixed 2 m behind the starting line. The HRD was applied to the subject at the waist level with a specially designed harness and belt (Figure 1). Sprint start was performed from standing split-stance position at the participants' own initiative. Backward swaying before the start was not allowed. Subjects were asked to sprint “all-out” at approximately 25 m distance. Between trials 5 to 10 min recovery was allowed. Two fastest sprint trials (t_0–20_) from the first visit were used for the within session reliability analyses, whereas their average was used for the between session and validity analyses (28).

### 2.4 Data acquisition and processing

#### 2.4.1 Timing gates

A single-beam photocell timing system (Brower Timing Systems, Draper, UT, USA) was used to acquire t_0–5_, t_0–10_, t_0–20_, t_5–10_, t_10–15_, and t_15–20_ with an accuracy of 0.01 s. Sensors were mounted on tripods above the hip height (∼120 cm above the floor level) to avoid undue interruption by the lower or the upper limbs (29). The pairs of timing gates were positioned at 0 m, 5 m, 10 m, 15 m, and 20 m distances with the first pair positioned 30 cm in front of the subject's front foot (i.e., actual start position) (30).

#### 2.4.2 Hydraulic resistance device

**Figure 2:**
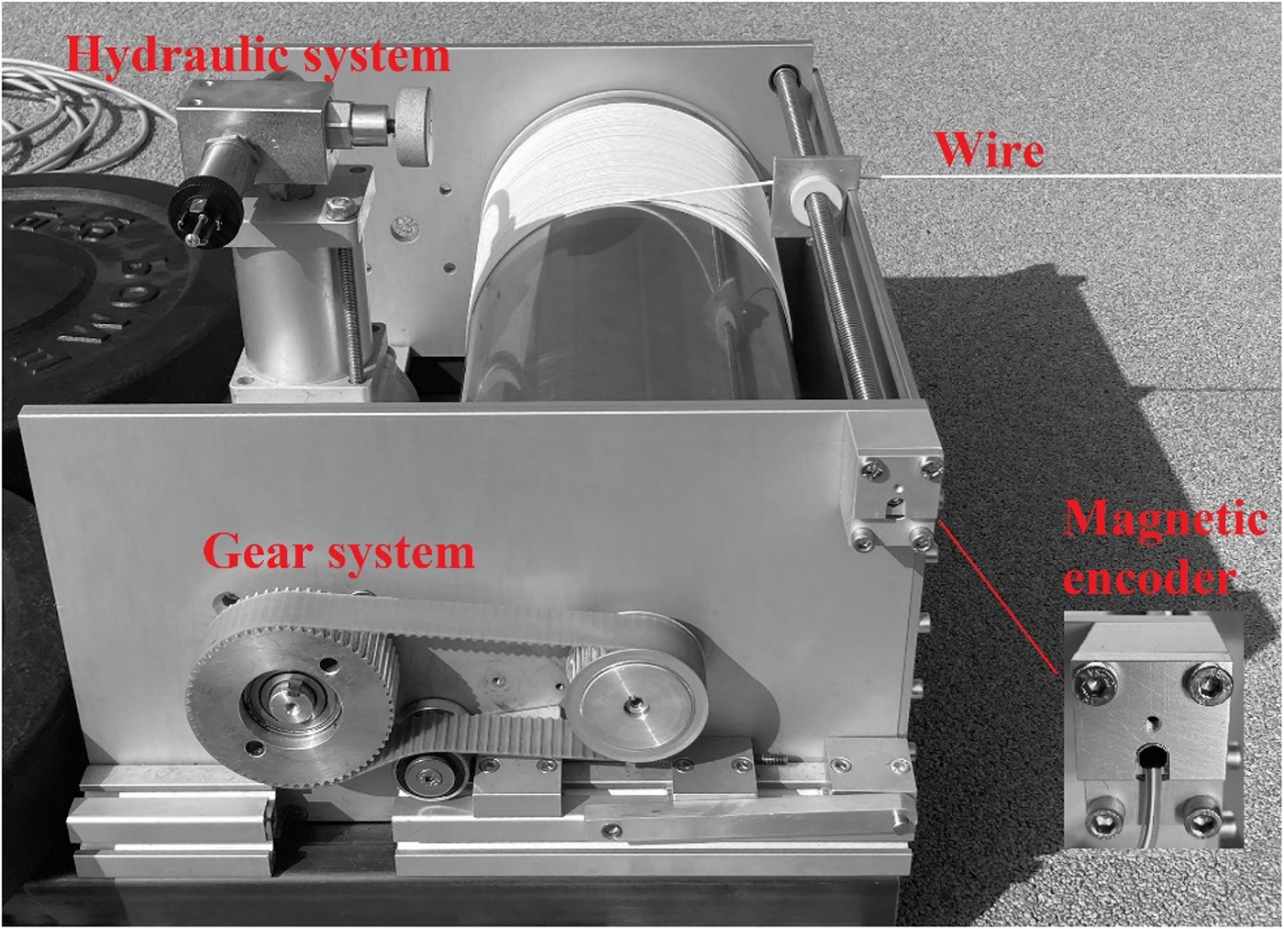
Hydraulic resistance device used to provide the resistance and assess resisted sprint performance.

**Figure 3:**
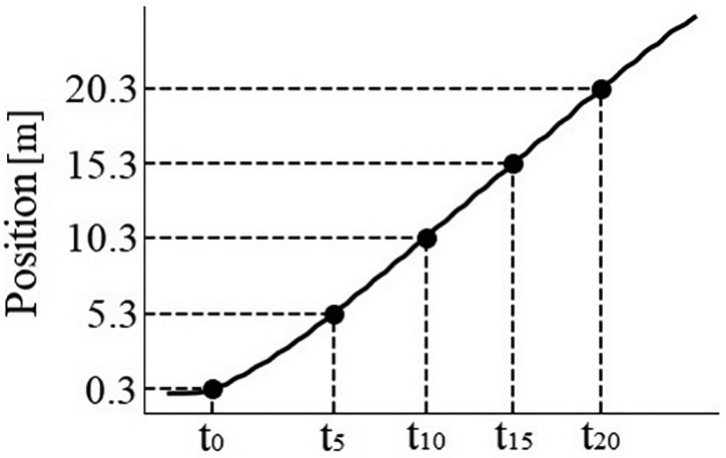
Split times data acquisition from the HRD signals. Graph represent the acquisition of split times for one subject. Split times were calculated from the position-time plots using 30-cm position delay to initialize sprint start (t_0_), time at 5.3-m distance (t_5_), time at 10.3-m distance (t_10_), time at 15.3-m distance (t_15_), and time at 20.3-m distance (t_20_) after the start.

The HRD, presented at Figure 2, is state-of-the-art device developed by the authors. It uses manually adjustable hydraulic system together with the gear system which were used to provide resistance via rope. For the purpose of this study, the resistance force was adjusted to four different loads based on laboratory calibration of the device. The magnetic incremental encoder (model AEAT-601B, Broadcom Inc., San Jose, CA, USA) mounted in the HRD allowed the measurement of subject's position and custom-developed software (ARS Dynamometry, S2P, Ljubljana, Slovenia; created in Labview 8.1., National Instruments, Austin, TX, USA) was used to acquire position signal at a frequency of 1,000 Hz. The raw signal was than processed using customized Python code, including the numpy, scipy, and pandas libraries, and filtered with a 4th order low-pass Butterworth filter (20 Hz cut-off frequency). The trigger criterion for time initiation of start was set at 30 cm from actual increase of position signal, corresponding to the distance between the first pair of timing gates and the starting line. Next, the t_0–5_, t_5–10_, t_10–15_, t_15–20,_ t_0–10_, and t_0–20_ were automatically determined from the position-time plots to obtain split times over 5, 10, and 20-m distances, respectively (see Figure 3).

### 2.5 Statistical analyses

Statistical analyses were performed in the SPSS version 26.0 (SPSS Inc, Chicago, IL, USA) and GraphPad Prism version 9.0.2 (GraphPad Software, Inc., San Diego, CA, USA). Mean and standard deviation (SD) were reported as measures of centrality and dispersion of the data. To test the assumption of normality the Shapiro-Wilk test was used for each data set. The two-sided intraclass correlation coefficient for single measures (ICC_3,1_), interpreted as poor (<0.50), moderate (0.50–0.74), good (0.75–0.90), and excellent (>0.90), was used to assess relative reliability (31). Absolute reliability was assessed using the typical error (TE; as standard deviation of the differences divided by √2) and coefficient of variation (CV; as typical error/mean × 100%). The following criteria were used to determine acceptable (CV ≤ 10%; ICC ≥ 0.80) and high (CV ≤ 5%; ICC ≥ 0.90) reliability (32). The standard error of measurement (SEM; SD × √1-ICC), and the minimal detectable change at the 95% confidence interval (MDC; SEM × √2 × 1.96) were calculated between sessions (33). Differences in HRD split times from first and second visit were assessed using paired sample *t*-test and Cohen's d effect size (ES). The ES was interpreted as trivial (<0.2), small (0.2–0.6), moderate (0.6–1.2), large (1.2–2.0) and very large (>2.0) (34). The validity of the HRD was based on the consistency and systematic bias between the timing gates and HRD, assessed by CV, two-sided intraclass correlation coefficient for average measures (ICC_3,k_), paired sample *t*-test, ES, and Bland-Altman plots with 95% limits of agreement. Statistical significance was set at *p* < 0.05.

## 3 Results

The Shapiro-Wilk test confirmed the normal distribution of the split times of the first and second session (*p* > 0.05). The reliability of the HRD split times within a session at different loads is shown in Table 2. The split times were highly reliable at all loads (ICC_3,1_ > 0.94; CV < 3.0%).

**Table 2:** Within session (*N* = 24) reliability of the hydraulic resistance device (HRD) for measuring split times.

HRD resistance		Trial 1	Trial 2	Relative reliability		Absolute reliability		
		Mean (SD)	Mean (SD)	ICC_3,1_ (95 CI)	Criteria	CV (95 CI)	TE (95 CI)	Criteria
Low	t_0–5_ [s]	1.20 (0.08)	1.22 (0.08)	0.97 (0.93–0.99)	Excellent	1.3 (1.0–1.8)	0.02 (0.02–0.01)	High
	t_5–10_ [s]	0.85 (0.06)	0.85 (0.06)	0.99 (0.99–1.00)	Excellent	0.6 (0.5–1.0)	0.01 (0.00–0.01)	High
	t_10–15_ [s]	0.76 (0.07)	0.76 (0.06)	0.99 (0.99–1.00)	Excellent	0.8 (0.6–1.1)	0.01 (0.01–0.01)	High
	t_15–20_ [s]	0.73 (0.06)	0.73 (0.06)	0.98 (0.95–0.99)	Excellent	1.3 (1.1–1.9)	0.01 (0.01–0.01)	High
	t_0–10_ [s]	2.05 (0.14)	2.07 (0.14)	0.99 (0.97–0.99)	Excellent	0.8 (0.7–1.2)	0.02 (0.01–0.02)	High
	t_0–20_ [s]	3.54 (0.27)	3.56 (0.26)	0.99 (0.98–1.00)	Excellent	0.7 (0.6–1.0)	0.03 (0.02–0.04)	High
Medium-low	t_0–5_ [s]	1.26 (0.10)	1.27 (0.09)	0.95 (0.96–0.98)	Excellent	1.7 (1.3–2.3)	0.02 (0.02–0.03)	High
	t_5–10_ [s]	0.90 (0.08)	0.90 (0.07)	0.98 (0.96–0.99)	Excellent	1.3 (0.9–1.6)	0.01 (0.01–0.01)	High
	t_10–15_ [s]	0.81 (0.08)	0.81 (0.07)	0.97 (0.94–0.98)	Excellent	3.0 (2.5–3.7)	0.02 (0.02–0.03)	High
	t_15–20_ [s]	0.78 (0.09)	0.78 (0.07)	0.98 (0.96–0.99)	Excellent	1.3 (1.0–1.8)	0.01 (0.01–0.01)	High
	t_0–10_ [s]	2.16 (0.17)	2.17 (0.16)	0.98 (0.95–0.99)	Excellent	1.2 (0.9–1.6)	0.03 (0.02–0.03)	High
	t_0–20_ [s]	3.76 (0.33)	3.76 (0.30)	0.98 (0.96–0.99)	Excellent	1.5 (1.2–2.1)	0.06 (0.04–0.08)	High
Medium-high	t_0–5_ [s]	1.28 (0.10)	1.27 (0.11)	0.96 (0.91–0.98)	Excellent	1.8 (1.4–2.5)	0.02 (0.02–0.03)	High
	t_5–10_ [s]	0.93 (0.09)	0.93 (0.09)	0.99 (0.98–1.00)	Excellent	0.9 (0.7–1.2)	0.01 (0.01–0.01)	High
	t_10–15_ [s]	0.84 (0.09)	0.84 (0.09)	1.00 (0.99–1.00)	Excellent	0.7 (0.5–0.9)	0.01 (0.01–0.01)	High
	t_15–20_ [s]	0.81 (0.09)	0.81 (0.10)	0.99 (0.99–1.00)	Excellent	0.9 (0.7–1.3)	0.01 (0.01–0.01)	High
	t_0–10_ [s]	2.21 (0.18)	2.20 (0.20)	0.98 (0.96–0.99)	Excellent	1.3 (1.0–1.8)	0.03 (0.02–0.04)	High
	t_0–20_ [s]	3.86 (0.36)	3.85 (0.38)	0.99 (0.98–1.00)	Excellent	0.9 (0.5–1.3)	0.04 (0.02–0.05)	High
High	t_0–5_ [s]	1.51 (0.17)	1.48 (0.17)	0.94 (0.88–0.97)	Good	2.9 (2.3–4.0)	0.04 (0.03–0.05)	High
	t_5–10_ [s]	1.17 (0.16)	1.17 (0.17)	0.99 (0.97–0.99)	Excellent	1.9 (1.5–2.6)	0.02 (0.02–0.03)	High
	t_10–15_ [s]	1.10 (0.18)	1.10 (0.18)	0.99 (0.98–1.00)	Excellent	1.7 (1.3–2.3)	0.02 (0.02–0.03)	High
	t_15–20_ [s]	1.11 (0.19)	1.13 (0.21)	0.98 (0.96–0.99)	Excellent	1.3 (1.0–1.8)	0.01 (0.02–0.02)	High
	t_0–10_ [s]	2.68 (0.32)	2.65 (0.33)	0.98 (0.96–0.99)	Excellent	1.9 (1.5–2.6)	0.05 (0.04–0.07)	High
	t_0–20_ [s]	4.90 (0.68)	4.88 (0.72)	0.99 (0.99–1.00)	Excellent	1.3 (1.0–1.7)	0.06 (0.05–0.08)	High

t_0–5_, sprint time at 0 to 5 m; t_5−10_, sprint time from 5 to 10 m; t_10−15_, sprint time from 10 to 15 m; t_15−20_, sprint time from 15 to 20 m; s, seconds; ICC_3,1_, intraclass correlation coefficient; TE, typical error; CV, coefficient of variation; SD, standard deviation; 95 CI, 95% confidence intervals.

As shown in Table 3, between session reliability of split times was high for most loads (ICC_3,1_ > 0.83; CV < 4.7), and trivial to small differences in timing were observed between sessions (ES < 0.49). The only exception was t_0–5_ at low load (ES = −0.64). The MDC of t_0–5_, t_0–10_, and t_0–20_ increased progressively with distance and HRD resistance and ranged from 3.2 to 9.9%.

**Table 3:** Between session reliability (*N* = 13) of the hydraulic resistance device (HRD) for measuring split times.

HRD resistance		Session 1	Session 2			Relative reliability		Absolute reliability			
		Mean (SD)	Mean (SD)	*Δ* in mean	ES	ICC_3,1_ (95 CI)	Criteria	CV (95 CI)	SEM	MDC (%)	Criteria
Low	t_0–5_ [s]	1.19 (0.08)	1.21 (0.08)	−0.015*	−0.64	0.97 (0.92–0.99)	Excellent	1.2 (0.9–1.9)	0.01	0.04 (3.2)	High
	t_5–10_ [s]	0.84 (0.06)	0.84 (0.06)	−0.004	−0.23	0.96 (0.88–0.99)	Good	1.6 (1.2–2.6)	0.01	0.03 (4.0)	High
	t_10–15_ [s]	0.74 (0.06)	0.75 (0.06)	−0.006	−0.49	0.97 (0.92–0.99)	Excellent	1.5 (1.1–2.3)	0.01	0.03 (3.9)	High
	t_15–20_ [s]	0.71 (0.06)	0.71 (0.06)	0.000	0.00	0.96 (0.88–0.99)	Excellent	1.9 (1.4–3.1)	0.01	0.03 (4.7)	High
	t_0–10_ [s]	2.05 (0.13)	2.05 (0.14)	−0.007	−0.11	0.91 (0.73–0.97)	Moderate	2.2 (1.6–3.5)	0.04	0.11 (5.3)	High
	t_0–20_ [s]	3.49 (0.24)	3.49 (0.26)	−0.017	−0.25	0.96 (0.88–0.99)	Good	1.6 (1.2–2.6)	0.05	0.14 (4.0)	High
Medium-low	t_0–5_ [s]	1.26 (0.07)	1.25 (0.09)	−0.004	−0.16	0.91 (0.73–0.97)	Moderate	2.2 (1.6–3.5)	0.02	0.07 (5.3)	High
	t_5–10_ [s]	0.90 (0.06)	0.88 (0.07)	0.005	0.33	0.84 (0.58–0.95)	Moderate	3.1 (2.2–4.9)	0.03	0.07 (8.1)	High
	t_10–15_ [s]	0.80 (0.06)	0.80 (0.07)	−0.002	−0.07	0.83 (0.54–0.94)	Moderate	3.6 (2.6–5.8)	0.03	0.07 (9.3)	High
	t_15–20_ [s]	0.78 (0.06)	0.76 (0.07)	0.005	0.26	0.84 (0.57–0.95)	Moderate	3.8 (2.7–6.1)	0.03	0.07 (9.4)	High
	t_0–10_ [s]	2.16 (0.13)	2.15 (0.16)	−0.010	−0.18	0.83 (0.55–0.94)	Moderate	3.0 (2.2–4.8)	0.06	0.17 (7.7)	High
	t_0–20_ [s]	3.74 (0.24)	3.70 (0.29)	0.004	0.07	0.96 (0.63–0.95)	Moderate	2.9 (2.1–4.7)	0.05	0.15 (3.9)	High
Medium-high	t_0–5_ [s]	1.25 (0.09)	1.26 (0.09)	−0.009	−0.28	0.95 (0.85–0.98)	Good	1.8 (1.3–3.0)	0.02	0.06 (4.5)	High
	t_5–10_ [s]	0.91 (0.08)	0.91 (0.08)	−0.002	−0.08	0.98 (0.93–0.99)	Excellent	1.4 (1.0–2.2)	0.01	0.03 (3.4)	High
	t_10–15_ [s]	0.82 (0.08)	0.83 (0.08)	−0.005	−0.28	0.99 (0.96–1.00)	Excellent	1.3 (0.9–2.0)	0.01	0.02 (2.7)	High
	t_15–20_ [s]	0.79 (0.09)	0.79 (0.08)	−0.002	−0.07	0.98 (0.93–0.99)	Excellent	1.8 (1.3–2.9)	0.01	0.03 (4.2)	High
	t_0–10_ [s]	2.17 (0.16)	2.18 (0.16)	−0.012	−0.26	0.97 (0.91–0.99)	Excellent	1.4 (1.0–2.3)	0.03	0.08 (3.5)	High
	t_0–20_ [s]	3.77 (0.33)	3.79 (0.32)	−0.019	−0.27	0.98 (0.94–0.99)	Excellent	1.3 (0.9–2.1)	0.05	0.13 (3.4)	High
High	t_0–5_ [s]	1.46 (0.14)	1.46 (0.15)	0.002	0.02	0.92 (0.77–0.97)	Good	3.1 (2.3–5.0)	0.04	0.11 (7.8)	High
	t_5–10_ [s]	1.14 (0.14)	1.13 (0.15)	0.010	0.17	0.94 (0.81–0.98)	Good	3.5 (2.5–5.6)	0.04	0.10 (8.7)	High
	t_10–15_ [s]	1.07 (0.15)	1.07 (0.16)	0.008	0.13	0.95 (0.84–0.98)	Good	3.8 (2.8–6.1)	0.04	0.10 (9.0)	High
	t_15–20_ [s]	1.08 (0.17)	1.07 (0.19)	0.012	0.16	0.93 (0.81–0.98)	Good	4.7 (3.4–7.5)	0.05	0.13 (12.3)	High
	t_0–10_ [s]	2.63 (0.28)	2.61 (0.3)	0.015	0.13	0.92 (0.78–0.97)	Good	3.3 (2.4–5.3)	0.08	0.23 (8.7)	High
	t_0–20_ [s]	4.75 (0.59)	4.72 (0.64)	−0.012	−0.09	0.94 (0.83–0.98)	Good	3.4 (2.5–5.5)	0.15	0.42 (9.9)	High

t_0–5_, sprint time at 0 to 5 m; t_5−10_, sprint time from 5 to 10 m; t_10−15_, sprint time from 10 to 15 m; t_15−20_, sprint time from 15 to 20 m; s, seconds; ICC_3,1_, intraclass correlation coefficient; CV, coefficient of variation; SD, standard deviation; SEM, standardized error of mean; MDC, minimal detectable change; 95 CI, 95% confidence intervals.

*p < 0.05.

**Figure 4:**
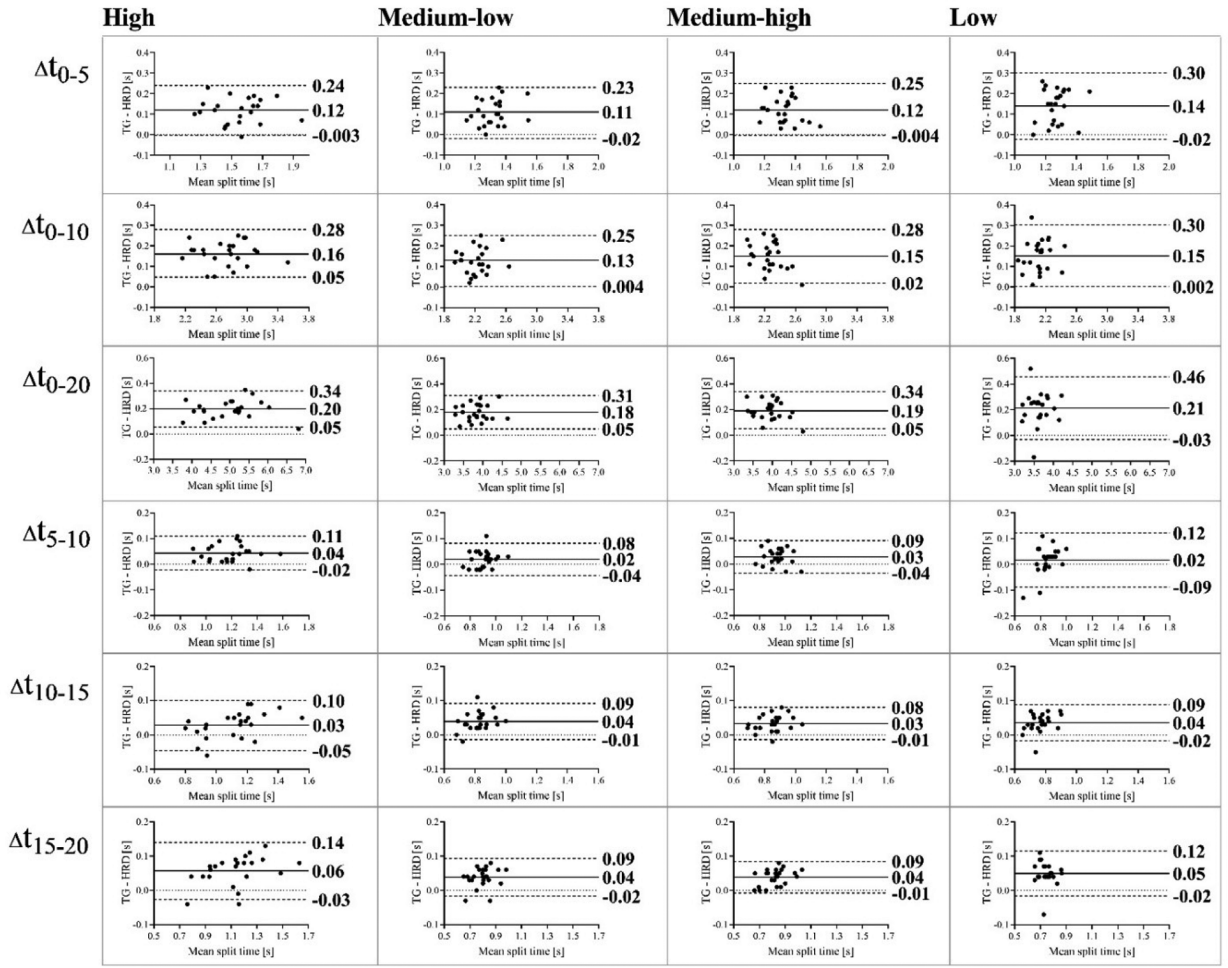
Bland-Altman plots showing absolute differences and 95% limits of agreement in split times (in seconds) between timing gates and the HRD (*Δ*t_0−5_, *Δ*t_0−10,_ *Δ*t_0−20,_ *Δ*t_5−10_, *Δ*t_10−15_, *Δ*t_15−20_) in sprints with high resistance, medium-low resistance, medium-high resistance, and low resistance. *Δ*t_0−5,_ absolute difference in split time between 0 and 5 m; *Δ*t_0−10_, absolute difference in split time between 0 and 10 m; *Δ*t_0−20_, absolute difference in split time between 0 and 20 m; *Δ*t_5−10_, absolute difference in split time between 5 and 10 m; *Δ*t_10−15_, absolute difference in split time between 10 and 15 m; *Δ*t_15−20_, absolute difference in split time between 15 and 20 m.

The validity of the HRD for measuring sprint split times at different loads is shown in Table 4. The consistency between timing gates and HRD split times was highest at high load (ICC_3,k_ > 0.92; CV < 2.7%) and lowest at low load (ICC_3,k_ > 0.47; CV < 4.4%). Regardless of the load, the consistency for t_0–5_ was always the lowest. The HRD systematically overestimated resisted sprint performance. In absolute values, t_0–5_, t_0–10_, and t_0–20_ were underestimated by 0.14–0.21, 0.12–0.19, 0.11–0.18, and 0.12–0.20 s at low, medium-low, medium-high, and high resistances, respectively. Bland-Altman plots 95% limits of agreement range for t_0–5_, t_0–10_, and t_0–20_ at all loads varied from minimum of 0.25 to maximum 0.49 s (Figure 4).

**Table 4:** Validity of the hydraulic resistance device **(**HRD) device for measuring split times (*N* = 24).

HRD resistance		TG in s	HRD in s	% *Δ* in mean	ICC_3,k_ (95 CI)	CV (95 CI)	ES	*p*
		Mean (SD)	Mean (SD)					
Low	t_0–5_ [s]	1.34 (0.10)	1.20 (0.09)	11.08	0.74 (0.41–0.89)	4.6 (3.6–6.5)	1.70	<0.001
	t_5–10_ [s]	0.86 (0.09)	0.84 (0.06)	2.01	0.86 (0.68–0.94)	4.4 (3.4–6.2)	0.32	0.131
	t_10–15_ [s]	0.79 (0.07)	0.75 (0.06)	4.67	0.96 (0.91–0.98)	2.5 (1.9–3.4)	1.34	<0.001
	t_15–20_ [s]	0.77 (0.06)	0.72 (0.06)	6.66	0.91 (0.80–0.96)	3.2 (2.5–4.4)	1.49	<0.001
	t_0–10_ [s]	2.20 (0.15)	2.04 (0.14)	7.18	0.93 (0.84–0.97)	2.6 (2.0–3.6)	1.99	<0.001
	t_0–20_ [s]	3.74 (0.28)	3.53 (0.27)	5.86	0.94 (0.87–0.98)	2.4 (1.9–3.4)	1.71	<0.001
Medium-low	t_0–5_ [s]	1.37 (0.11)	1.26 (0.09)	8.27	0.89 (0.75–0.95)	3.4 (2.7–4.8)	1.70	<0.001
	t_5–10_ [s]	0.91 (0.08)	0.89 (0.08)	2.17	0.96 (0.90–0.98)	2.5 (2.0–3.5)	0.61	0.007
	t_10–15_ [s]	0.84 (0.08)	0.80 (0.08)	4.71	0.97 (0.93–0.99)	2.3 (1.8–3.2)	1.44	<0.001
	t_15–20_ [s]	0.82 (0.09)	0.78 (0.08)	4.76	0.97 (0.94–0.99)	2.5 (1.9–3.5)	1.36	<0.001
	t_0–10_ [s]	2.28 (0.18)	2.16 (0.17)	5.67	0.97 (0.92–0.99)	2.0 (1.5–2.8)	2.02	<0.001
	t_0–20_ [s]	3.93 (0.34)	3.75 (0.33)	4.62	0.99 (0.98–1.00)	1.2 (0.9–1.7)	2.70	<0.001
Medium-high	t_0–5_ [s]	1.39 (0.09)	1.27 (0.11)	9.12	0.89 (0.74–0.95)	3.4 (2.7–4.8)	1.89	<0.001
	t_5–10_ [s]	0.95 (0.08)	0.92 (0.09)	2.98	0.96 (0.91–0.98)	2.4 (1.9–3.4)	0.87	<0.001
	t_10–15_ [s]	0.86 (0.09)	0.83 (0.09)	3.89	0.98 (0.96–0.99)	2.0 (1.6–2.8)	1.37	<0.001
	t_15–20_ [s]	0.84 (0.10)	0.80 (0.09)	4.66	0.98 (0.97–0.99)	2.0 (1.6–2.8)	1.63	<0.001
	t_0–10_ [s]	2.34 (0.17)	2.19 (0.19)	6.57	0.96 (0.92–0.98)	2.1 (1.6–2.9)	2.23	<0.001
	t_0–20_ [s]	4.04 (0.35)	3.84 (0.37)	4.94	0.99 (0.98–1.00)	1.3 (1.0–1.9)	2.64	<0.001
High	t_0–5_ [s]	1.60 (0.17)	1.48 (0.17)	7.72	0.96 (0.92–0.98)	2.8 (2.2–4.0)	1.92	<0.001
	t_5–10_ [s]	1.20 (0.17)	1.16 (0.16)	3.63	0.99 (0.98–1.00)	2.0 (1.5–2.8)	1.29	<0.001
	t_10–15_ [s]	1.10 (0.18)	1.12 (0.20)	2.63	0.99 (0.98–1.00)	2.4 (1.9–3.4)	0.76	0.001
	t_15–20_ [s]	1.17 (0.22)	1.11 (0.20)	4.99	0.99 (0.98–1.00)	2.7 (2.1–3.7)	1.32	<0.001
	t_0–10_ [s]	2.81 (0.33)	2.64 (0.33)	5.94	0.99 (0.98–1.00)	1.5 (1.2–2.1)	2.79	<0.001
	t_0–20_ [s]	5.09 (0.73)	4.89 (0.73)	3.92	1.00 (0.99–1.00)	1.0 (0.8–1.4)	2.72	<0.001

TG, timing gates; HRD, hydraulic resistance device; t_0–5_, sprint time at 0 to 5 m; t_5−10_, sprint time from 5 to 10 m; t_10−15_, sprint time from 10 to 15 m; t_15−20_, sprint time from 15 to 20 m; s, seconds; ICC_3,k_, intraclass correlation coefficient; CV, coefficient of variation; SD, standard deviation; ES, Cohen's d effect size; 95 CI, 95% confidence intervals.

## 4 Discussion

The aim of this study was to assess the validity and reliability of the HRD for the evaluation of resisted sprint time. The split times measured with HRD were highly reliable within and between sessions. Despite moderate to excellent consistency between the timing gates and HRD, split times were systematically underestimated. These results suggest that the HRD could be used to record resisted sprint split times under various loads.

The high within session reliability of the HRD is consistent with other studies that have investigated different resistance devices. Godwin et al. (35) measured the split times of resisted sprints with Run Rocket™ using a radar gun and found good to excellent intrasession reliability for the 5 and 10 m times at two resistance levels (ICC = 0.79–0.98; CV = 2.0%–4.6%). In our study, HRD split times provided even higher within session reliability, both in absolute and relative terms (ICC > 0.94; CV < 2.9%). As these results are also comparable to the reliability of a valid motorized resistance device in sprints with 50 N, 80 N and 110 N resistance (18), we can confirm our initial hypothesis of high reliability of the HRD for timing of resisted sprints.

The reliability of magnetic incremental encoders for monitoring performance during sprints with resisted is still relatively unexplored. Highly valid and reliable devices that measured sprint times without resistance at 5, 10, 20 and 30 m distances provided ICC, CV, TE and SWC in the range of 0.83–0.97, 0.8–2.10%, 0.03–0.08 s and 0.01–0.04 s, respectively (36, 37). However, resisted sprints cannot be directly compared with sprints without resistance. Compared to radar used in the study by Godwin et al. (35), HRD showed similar between session reliability for timing resisted sprints (ICC = 0.87–0.97; CV = 2.0%–4.1% for radar vs. ICC_3,1_ = 0.83–0.99; CV = 1.2%–4.7% for HRD). These results also confirm that HRD is reliable for assessing sprint performance between sessions, therefore the MDC values for the split times are shown in Table 3. For example, at the medium-high load, the t_0–10_ improvement of 3.5% can be identified as a true change in performance beyond the HRD measurement error. This is comparable to the MDC of the 10 m sprint time using the radar gun (28). Therefore, the HRD can be used to monitor changes in resisted sprint performance.

The validity of the HRD for measuring performance in sprints with resistance is an important feature. The HRD indeed showed excellent reliability, but systematically underestimated the split times compared to the timing gates. Similar observations were previously made by Raković et al. (18), who found that the observed bias was due to the starting position, which can contribute to large absolute differences in split times (38). As the first 5 m of the sprint reflects the start itself, it is not surprising that the largest relative differences for t_0–5_ across all HRD loads were observed at low load (i.e., from 7% to 11%). In absolute terms, the HRD bias for t_0–5_, t_0–10_ and t_0–20_ remained relatively constant among loading conditions, ranging from 0.11 to 0.21 s. Similar to Raković et al. (18), differences in the initiation of the start between HRD and timing gates are likely responsible for the observed underestimation. As the photocell height of 1.2 m is typically higher than waist level on average, changes in trunk angle from a crouched start to a more upright position later in the sprint may have increased the displacement in HRD and resulted in shorter sprint times. This could systematically underestimate split times when using HRD. Despite the standardization of the sprint start, there was still some biological variability in the start position between subjects causing the variation in the distance between the HRD harness and the first pair of timing gates. Consequently, the Bland-Altman 95% limits of agreement intervals (individual agreement) of t_0–5_, t_0–10_, and t_0–20_ were greater than the MDC of the HRD split times. Given the observed good to excellent consistency between HRD and TG for t_0–10_ and t_0–20_ (lower 95% CI for ICC ranged from 0.84 to 0.99), the HRD appears to be valid for assessing resisted sprint split times, particularly at longer distances. Thus, the HRD device can be independently used in practice to measure resisted sprint split times and consequently monitor sprint performance.

Although the results of this study are promising, some of the limitations should be emphasized. First of all, the study was conducted on student athletes, so the result cannot be generalized to the population of elite athletes such as sprinters, footballers or rugby players. In order to obtain population-specific MDC, future studies should investigate the between session reliability of HRD for assessing resisted sprint performance in a group of elite athletes who frequently perform this type of sprint as part of their training regime. Second, we included a wide range of amateur athletes from individual and team sports where acceleration and resisted sprints are part of the sport-specific movement or training. This heterogeneity, in turn, could seemingly increase the consistency between HRD and timing gates. Finally, using single beam timing gates as a criterion may not be the best option when assessing the validity of HRD for evaluating the performance of resisted sprints and future studies should evaluate the validity of HRD together with gold-standard systems.

In conclusion, the results of this study show that the HRD provides good within and between session reliability to monitor split times during resistance sprinting. Regardless of high systematic bias observed between the HRD and single-beam timing gates, very high consistency between methods confirmed good validity of the HRD for measuring sprint times over 10 and 20-m distances. Therefore, we conclude that HRD can be used as a training and testing tool by coaches, athletes and sports scientists for training and research purposes. However, the MDC and underestimation of split times should be taken into account.

## Data Availability

The raw data supporting the conclusions of this article will be made available by the authors, without undue reservation.
